# Triage of Women Testing Positive With the *care*HPV Test on Self-Collected Vaginal Samples for Cervical Cancer Screening in a Low-Resource Setting

**DOI:** 10.1200/JGO.2016.008078

**Published:** 2017-09-29

**Authors:** Usha Rani Poli, Swarnalata Gowrishankar, Meenakshi Swain, Jose Jeronimo

**Affiliations:** **Usha Rani Poli**, MNJ Institute of Oncology & Regional Cancer Center; **Swarnalata Gowrishankar** and **Meenakshi Swain**, Apollo Hospitals, Hyderabad, India; and **Jose Jeronimo**, PATH, Seattle, WA.

## Abstract

**Purpose:**

Human papillomavirus (HPV) DNA screening reduces cervical cancer incidence and mortality in low-resource settings. Self-collected vaginal samples tested with affordable HPV tests such as *care*HPV can increase the rate of screening in resource-constrained settings. We report the role of visual inspection with acetic acid (VIA) as a triage test for women testing positive with the *care*HPV test on self-collected vaginal samples.

**Methods:**

As part of a multicountry demonstration study, 5,207 women 30 to 49 years of age were recruited from urban slums to undergo four cervical screening tests using the *care*HPV test on self-collected vaginal samples, provider-collected cervical samples, the Papanicolaou test, and VIA. All women who tested positive for any of the screening tests were evaluated with colposcopy and guided biopsies, followed by treatment if any cervical lesions were detected. The data from the 377 women who tested positive for HPV in the self-collected vaginal samples were also analyzed to assess the performance of VIA, conventional cytology, and colposcopy, as triage tests in the detection of cervical cancer and precancerous lesions.

**Results:**

Nineteen percent of women who tested positive for vaginal HPV (V-HPV) also tested positive with the VIA test; cervical intraepithelial neoplasia 2+ lesions were detected in 58% of these women. In the 30 % of the women who tested positive for V-HPV with cytology triage, cervical intraepithelial neoplasia 2+ lesions were detected in 80% of these women. The colposcopy referrals for women who tested positive for V-HPV were reduced from 7.6% to 1.5% by VIA triage, and to 2.3% by cytology triage. Although the sensitivity was reduced, the positive predictive value improved after triage with VIA and cytology.

**Conclusion:**

This study reflects the optimal role of VIA triaging for treatment selection of lesions among those who test positive for V-HPV in screen and treat screening programs that use an HPV test in low-resource settings.

## INTRODUCTION

Approximately one fourth of the world’s burden of cervical cancer is in India, with122,844 new cases and 67,477 deaths as a result of cervical cancer reported in the year 2012.^1^ Despite multiple efforts, cervical cancer continues to be a major public health problem.

Screening services are inadequate in remote and rural areas, and cytology-based screening test performance is suboptimal. Cervical cytology (the Papanicolaou [Pap] test) has been implemented widely in developed countries, but it is not suitable for areas with limited resources because of the test's complexity, the lack of cytotechnologists to review the slides, and the test’s suboptimal sensitivity.^2^ Various new alternative strategies suitable for low-resource settings have been evaluated to replace cytology. Visual inspection with acetic acid (VIA)—a simple test that can be performed easily by trained mid-level health care providers—is an optimal screening method for a single-visit screen-and-treat approach for resource-constrained countries. In a recent meta-analysis, the sensitivity of VIA for the detection of high-grade cervical lesions ranged from 41% to 92% because of the test's subjectivity.^3^ Similarly, specificity ranged from 49% to 98%.^3^ The human papillomavirus (HPV) DNA test is a more objective and sensitive test than VIA and cytology, and it is suitable for population-based screening in India.^4^ A single round of HPV DNA screening is associated with a reduction in cervical cancer incidence and mortality in low-resource settings.^5^

One affordable, simple, and rapid HPV DNA test on the market is the *care*HPV test.^6^ Self-collection of vaginal samples for HPV DNA testing is acceptable to women^7^ and is effective for the detection of high-grade cervical lesions.^4^ A strategy that is based on self-collected vaginal samples for HPV testing could increase access to cervical screening and improve population coverage, especially in rural and remote areas. Because women who test negative for HPV have a negligible risk of developing cervical cancer over a 5- to 10-year period, screening intervals may be increased significantly in women with a negative HPV screening result.^8^ A positive HPV test indicates the presence of infection with any of the 14 types of high-risk oncogenic HPV types. However, the specificity of the test is not optimal because of the occurrence of transient infections without any cervical lesions. Therefore, the management of women who screen HPV-positive requires a triage test to identify and diagnose the precancerous or cancerous lesions requiring treatment. The inclusion of a second test for triaging women whose primary screen is HPV-positive in screening protocols may also reduce unnecessary referrals for diagnosis and treatment, which could decrease the burden on the weak health care systems in areas with limited resources.

Screening protocols from wealthier countries recommend using HPV testing as the primary screening test and cytology as the triage test to improve specificity.^9^ WHO guidelines recommend several options for primary screening, including HPV genotyping, cytology, or VIA triage for follow-up of women who test positive for HPV infection.^10^ However, using VIA as a triage option for primary HPV testing has not been investigated extensively in field studies in developing countries. The *care*HPV test has been evaluated in comparison with other screening tests in a real-life field setting in the urban slums of Hyderabad (the Screening Technologies to Advance Rapid Testing–Utility and Program Planning [START-UP] project) in South India.^4^ Using data from this community-based cervical screening program, we report on the performance of VIA and cytology as triage tests in the detection of cancer and precancers in women who test positive with the *care*HPV DNA test using self-collected vaginal samples.

## METHODS

As a part of the international START-UP project, a comparative evaluation of the *care*HPV test with VIA and Pap smears to detect cervical cancer and precancerous lesions was conducted in Hyderabad, India, from January 2010 to December 2013. This study was approved by institutional ethics committees of the MNJ Institute of Oncology and the Program for Appropriate Technologies in Health (PATH), in the United States. The comparative results of all four screening tests in the detection of cervical intraepithelial neoplasia (CIN) 2+ lesions have been published.^4^ The data from 377 women who tested positive for vaginal HPV (V-HPV) in this study were also analyzed to evaluate the performance of VIA, cytology, and colposcopy as triage tests in the detection of CIN 2+ lesions.

Five thousand two hundred seven women, from 30 to 49 years of age, were recruited from the general population residing in urban slums to undergo cervical cancer screening. The participants were married, non-pregnant women with an intact uterus. They had not been diagnosed previously with cervical cancer or precancer, were willing to undergo screening, and were able to give informed consent. Local community motivators conducted health education sessions and group discussions before the cervical screening took place. Women were invited for screening either in the outreach community screening clinic or in the makeshift mobile screening camps near their localities. All eligible women who consented were offered four screening tests during the same screening visit. First, they were instructed by the health worker on how to take a self-collected vaginal sample for *care*HPV testing in privacy using a soft-bristled brush. The samples were transported in Digene collection media (QIAGEN, Venlo, the Netherlands). They then underwent speculum examination to collect cervical samples for the *care*HPV test. A Pap test and VIA were performed sequentially on all the women by trained health workers. Women with positive results on any of the screening tests underwent colposcopy and guided biopsies for histopathologic confirmation by a trained medical officer. Women who tested VIA-positive underwent colposcopy and biopsy in the same visit, but women who were HPV- and cytology-positive were informed and later examined after the results became available, which ranged from 1 to 4 weeks. Biopsy specimens were taken from colposcopically abnormal areas or at the 12 o'clock position if there was no visible acetowhite lesion. Women with confirmed CIN grade 2+ lesions were either treated with cryotherapy at the local clinic or referred to a hospital for additional evaluation and treatment.

Vaginal and cervical samples for *care*HPV testing were transported to the base hospital and stored at room temperature (15°C to 30°C) for a maximum of 14 days, at 2°C to 8°C for a maximum of 30 days, or at −20°C for a maximum of 60 days. The *care*HPV test results were quantified as a ratio of viral load (expressed in relative light units) to the mean relative light units from a positive control set at a 1 pg/mL cutoff. The equipment had been set by the manufacturer to call a sample positive if the ratio was ≥ 1.0 pg/mL. A local pathologist who was aware that the samples were part of a study to compare multiple screening options examined the Pap test samples, maintaining quality assurance. The samples were evaluated according to the Bethesda classification system; any smear with atypical squamous cells of undetermined significance or more severe changes was considered positive. Women with the appearance of acetowhite areas in the transformation zone with 4% acetic acid were classified as VIA positive by the trained health workers.

Data entry and analysis were performed using Stata software, version 12.0 (StataCorp, College Station, TX). Sensitivity, specificity, predictive values, and 95% CIs for the screening tests were calculated using two-by-two tables and standard formulas.

## RESULTS

Out of the 5,207 women recruited for screening, self-collected or vaginal samples for *care*HPV testing were collected from 4,947 women. Samples were self-collected by 85% of these women. Twenty-four participants with inadequate samples and eight with missing samples were excluded from the analysis.

**Fig 1:**
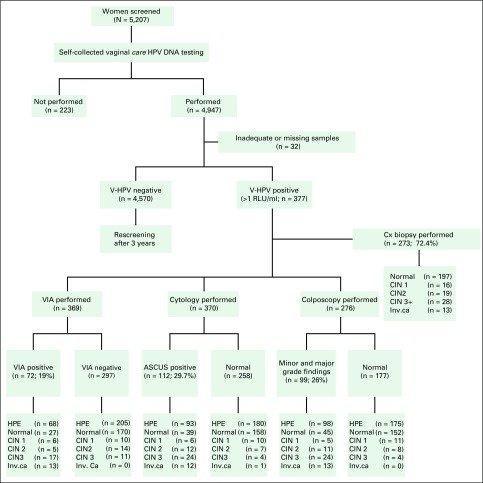
Study flowchart showing triage tests and diagnosis of women who test positive for vaginal human papillomavirus (V-HPV). ASCUS, atypical squamous cells of undetermined significance; CIN, cervical intraepithelial neoplasia; Cx, cervix; HPE, histopathologic examination; HPV, human papillomavirus; Inv. ca, invasive cancer; RLU, relative light units; VIA, visual inspection with acetic acid.

The *care*HPV test for V-HPV was positive in 377 women. The mean age of the women who were V-HPV positive was 36.3 years (standard deviation, 6.19 years), and 15% were postmenopausal (Table 1).The 4,570 women who tested negative for V-HPV were advised to wait 3 years for rescreening because they were at negligible risk in the near future (Fig 1). Seventy-two women (19%) tested positive using VIA, 112 women (29%) tested positive with cytology, and 99 women (26%) had colposcopically minor- and major-grade abnormalities. Colposcopies and guided biopsies were performed in 273 women (72%) who were V-HPV positive. Among the quarter of those women who did not get colposcopic confirmation, approximately 95% were VIA negative and 80% were cytology negative. Overall, among the women who tested positive for V-HPV, 16 had CIN 1, 19 had CIN 2, and 41 had CIN 3+ histologically confirmed lesions. The outcome measures for the triage testing were calculated for the 273 women with biopsy results (Table 2).

**Table 1:**
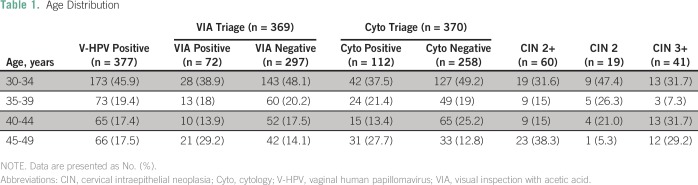
Age Distribution

**Table 2:**
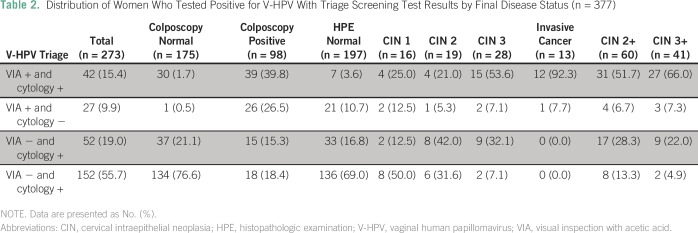
Distribution of Women Who Tested Positive for V-HPV With Triage Screening Test Results by Final Disease Status (n = 377)

Approximately 52% of all high-grade lesions (CIN 2+) among the women who were V-HPV positive were detected by both VIA and cytology triage, but 13% were missed by both tests. VIA triage alone detected 58% of CIN 2+ and 73% of CIN 3+ lesions, whereas cytology detected 80% and 88%, respectively. VIA detected all 13 invasive cancers, but cytology missed one (7.7%; Table 3). Only 72% of women who tested positive for V-HPV were examined colposcopically. Colposcopy detection of CIN 2+ was similar to that of cytology triage detection (Table 3). Colposcopy detected 22% of CIN 2+ lesions that were missed by VIA. Although 74% of grade 2 CIN lesions were not detected by VIA triage, both colposcopy and cytology triaging also missed 40% of CIN grade 2 lesions. Most of the women with undetected CIN 2 lesions were younger women (Table 1). VIA triage led to the referral of 19% of the women who tested positive for V-HPV for diagnosis and treatment, which resulted in the detection of 58% of CIN 2+ lesions. Quality-assured cytology triage resulted in the referral of 30% of the women who tested positive for V-HPV, with 80% detection of CIN 2+ lesions; however, cytology required another visit for diagnostic confirmation. In contrast, VIA results were available immediately during the same visit. The colposcopy referrals of women who tested positive for V-HPV would thus be reduced from 7.6% to 1.5% by VIA triage, and to 2.3% by cytology triage. Even though the sensitivity at the CIN 2+ threshold was reduced with both VIA and cytology triaging, the positive predictive value (PPV) improved greatly (Table 2).

**Table 3:**
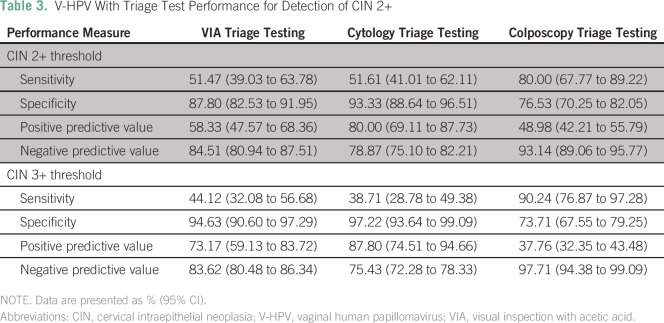
V-HPV With Triage Test Performance for Detection of CIN 2+

## DISCUSSION

With the emergence of HPV DNA testing as a primary screening modality, a promising and affordable new test, *care*HPV, is available for cervical cancer prevention in low- and middle-income countries. Our study reflects the data from a real-life limited resource setting. If *care*HPV testing on self-collected vaginal samples is used as a primary screening test in a population such as that in the low socioeconomic slums of Hyderabad where it is readily acceptable, only a limited number of women would be referred for additional evaluation, and most CIN2+ lesions would be detected. Self-sampling has the advantage of being better accepted because it does not require a pelvic examination and thus improves screening coverage.^10^ Although approximately 5,000 women were screened in our study, only 377 women (7.5%) who were V-HPV positive would be referred to the health facility for a pelvic evaluation, which is a more manageable number when resources are limited. In resource-constrained settings, a primary HPV screening method with low PPV would lead to excessive referrals of women with transient or insignificant HPV infections, and to the treatment of nonprogressive cervical lesions, resulting in overtreatment if used as a screen-and-treat method. Because triaging of these women with quality-assured cytology or genotyping may not be possible because of cost implications, alternatives such as VIA should be the choice. The WHO expert panel also recommends VIA triage of women who test positive for HPV as a screen-and-treat strategy in low- and middle-income countries, especially where resources are limited or quality assurance is not maintained for cytology programs.^11^

In our study, the detection rate of CIN 2+ lesions was similar with both cytology and colposcopy triage and was higher than that with VIA triaging. The colposcopy referral rate would be reduced to six times in VIA triage and five times in cytology triage for these women at risk. A limitation of our study is that all the women who tested positive for V-HPV did not undergo diagnostic confirmation, and it was also observed that 80% and 95% of these women were cytology and VIA negative, respectively. In addition, approximately 17% of women who were cytology-positive and who required colposcopy did not undergo diagnostic confirmation because there was a waiting period of 3 to 4 weeks for cytology results. In this study, VIA triaging of women who tested positive for V-HPV were detected by primary HPV testing alone with a sensitivity of 51.47% (Table 3). Muwonge et al,^12^ in a study in India, observed that detection rates of CIN 2+ lesions were similar when women who tested positive for HPV were triaged with either cytology or VIA triage tests. VIA triage significantly improved the PPV, which was comparable to that of cytology triaging, and it also reduced the colposcopy referral rate by 59%; however, it missed 18% of CIN 2+ lesions.^12^

It is crucial that the triaging strategy not reduce the sensitivity of the primary screening test. The sensitivity of detection of CIN 3+ lesions with VIA triage in our study was also reduced significantly because of its questionable usefulness. Moreover, VIA or cytology triage improved the PPV. In another large HPV-based screening program conducted in West Bengal, India, VIA triaging of women who tested positive for HPV using provider-collected cervical samples improved the PPV for detection of CIN 3+ lesions by almost 30%.^13^ The loss of sensitivity after triaging was high in this study as well, and the VIA triage missed 31.6% of the CIN 3+ lesions detected originally by the HPV test alone.^13^ Similar observations were also found in a randomized controlled trial from sub-Saharan Africa that used self-collected samples.^14,15^

Although VIA triage in our study failed to detect one half of the high-grade lesions, it effectively detected three quarters of the CIN 3+ lesions and all invasive cancers that required immediate treatment. If VIA were to be used to triage women who are HPV positive and to determine who should be referred for additional treatment and for treatment selection, as in our study, 72 women would have been selected for additional diagnostic evaluation and treatment. Overall, 40% of the women with CIN2+ lesions that were detected originally by primary HPV screening would be missed for treatment by VIA triage. In contrast, a high-quality Pap test triage such as that used in our study would miss 21% of the CIN 2+ cases detected by HPV testing alone. The lesions missed by VIA triage included only 27% of lesions at the CIN 3+ threshold and 74% of CIN grade 2 lesions. Therefore, VIA triage effectively detects most CIN 3 lesions and all invasive cancers but fails to detect the majority of CIN 2 lesions. Most of these CIN grade 2 lesions undetected by VIA triage could be either nonprogressing or regressive lesions requiring close follow-up because V-HPV positivity indicates a higher risk of developing neoplasia.^16,17^ VIA triage should be preferred in low-resource areas because the necessary consumables like acetic acid are readily available, trained nurses can perform the test, and the results are available immediately, which would allow triage and treatment in a single visit.

Hence, if VIA were to be used to triage women who test positive for HPV, it could determine who should be referred for additional treatment and for treatment selection of high-grade cervical lesions. Therefore, VIA triage could be considered an optimal component of a screen-and-treat strategy in limited settings where women who are VIA positive can receive immediate treatment, and in which self-sampling, combined with an affordable and field-friendly HPV test, is used as the primary cervical screening method. Adopting this strategy would not only improve screening coverage, but also benefit women at risk by allowing treatment in the same visit, minimizing the loss of these women at risk to follow-up and treatment delays because of the need for multiple visits.^18^ Large prospective studies using this strategy with longitudinal follow-up will be required to establish its effectiveness in low- and middle-income countries.
